# Patient-related Factors Associated with Potentially Unnecessary Transfers for Pediatric Patients with Asthma: A Retrospective Cohort Study

**DOI:** 10.5811/westjem.18399

**Published:** 2024-04-09

**Authors:** Gregory A. Peters, Rebecca E. Cash, Scott A. Goldberg, Jingya Gao, Lily M. Kolb, Carlos A. Camargo

**Affiliations:** * Harvard Medical School, Boston, Massachusetts; † Massachusetts General Hospital, Department of Emergency Medicine, Boston, Massachusetts; ‡ Brigham and Women’s Hospital, Department of Emergency Medicine, Boston, Massachusetts; § The Donald and Barbara Zucker School of Medicine at Hofstra/Northwell, Hempstead, New York; ∥ Harvard T.H. Chan School of Public Health, Department of Epidemiology, Boston, Massachusetts

## Abstract

**Background/Objective:**

Asthma is a common chronic medical condition among children and the most common diagnosis associated with interfacility transports for pediatric patients. As many as 40% of pediatric transfers may be unnecessary, resulting in potential delays in care and unnecessary costs. Our objective was to identify the patient-related factors associated with potentially unnecessary transfers for pediatric patients with asthma.

**Methods:**

We used patient care data from the California Department of Health Care Access and Information patient discharge and emergency department (ED) datasets to capture ED visits where a pediatric patient (age 2–17 years) presented with asthma and was transferred to another ED or acute care hospital. The outcome of interest was a potentially unnecessary transfer, defined as a visit where length of stay after transfer was <24 hours and no advanced services were used, such as respiratory therapy or critical care. Patient-related characteristics were extracted, including age, gender, race/ethnicity, primary language, insurance status, and clinical characteristics. First, we used descriptive statistics to compare necessary vs unnecessary transfers. Second, we used generalized estimating equations accounting for clustering by ED to estimate odds ratios (OR) and identify factors associated with potentially unnecessary transfers.

**Results:**

A total of 4,233 pediatric ED patients were transferred with a diagnosis of asthma, including 461 (11%) transfers that met criteria as potentially unnecessary. Median age was 12 years (interquartile range 7–15), and 46% were female. Factors associated with increased odds of potentially unnecessary transfer while controlling for key factors included younger age (eg, 2–5 years, OR 2.0, 95% confidence interval [CI] 1.4–2.9), male gender (OR 1.4, 95% CI 1.1–1.7), and Hispanic ethnicity (OR 1.6, 95% CI 1.2–2.1), while multiple hospitalizations for asthma per year was associated with decreased odds (OR 0.2, 95% CI 0.1–0.4).

**Conclusion:**

Several patient-related factors were associated with increased or decreased odds of potentially unnecessary transfers among pediatric patients presenting to the ED with asthma. These factors can be considered in future work to better understand, predict, and reduce unnecessary transfers and their negative consequences.

Population Health Research Capsule What do we already know about this issue?

*Asthma is the most common diagnosis associated with interfacility transfer of pediatric patients. Transfers entail costs, delays in care, and resource strain.*

What was the research question?

*Which patient-related factors are associated with unnecessary transfer for pediatric asthma exacerbations?*

What was the major finding of the study?

*Younger age (OR 2.0, 95% CI 1.4-2.9) and Hispanic ethnicity (OR 1.6, 95% CI 1.2–2.1) were associated with unnecessary transfer.*

How does this improve population health?

*Several patient-related factors were associated with increased odds of unnecessary transfer, which can cause preventable strain on families and healthcare systems.*

## INTRODUCTION

### Background

Asthma is a common chronic medical condition among children,^1^ affecting 7.5% of the overall pediatric population^2^ with peak prevalence in young teenagers (12–14 years) at nearly 11%.^3^ Children with asthma exacerbations account for approximately 650,000 emergency department (ED) visits in the US annually, and many of these visits result in hospital admission, including via interfacility transfer by emergency medical services (EMS) to another hospital.^4^ Indeed, asthma is the most common primary medical diagnosis associated with interfacility transport for pediatric patients.^5^ Interfacility transfers are typically initiated by emergency physicians, citing a need for a higher level of care (ie, critical care), recommendation of specialty services (eg, pediatric pulmonology), or capacity-related limitations (ie, current availability of beds or other resources). Despite the commonplace nature of pediatric transfers for asthma in the ED, there is no prior literature to support policy makers and other stakeholders that include emergency physicians and administrators when navigating this routine decision-making process.

### Importance

Prior work has shown that more than 40% of undifferentiated pediatric transfers were either discharged directly from the receiving ED or within 24 hours of direct admission upon transfer,^6^ and that only one-quarter of all pediatric transfers are completed to provide a higher or more specialized level of care to the patient.^7^ These outcomes are important because interfacility transfers are associated with missed doses of medication, prolonged time to initiation of inpatient care, and a substantial financial burden on families and taxpayers.^7^^,^^8^ With these risks and costs in mind, a recent study of 1.7 million pediatric transfers in the US reported that only 12.3% of all pediatric transfers met criteria for a medically necessary transfer, demonstrating the limited direct benefit to patient care in many cases retrospectively.^9^ Moreover, socioeconomically vulnerable populations are disproportionally affected by asthma,^10^^–^^14^ and the disproportionate financial burden of interfacility transfers on underserved rural patients has been previously described,^8^ indicating the important likely health equity implications of this topic. These considerations underscore the need for improved guidance for policy makers when contemplating the routine practice of interfacility transfer of pediatric patients who present to the ED with asthma.

### Goals of this Investigation

We aimed to describe the patient-related factors associated with potentially unnecessary interfacility transfer of pediatric patients presenting to the ED with asthma. Our ultimate goal in this work is to stimulate discussion and future research regarding the characteristics of patients most likely to experience the consequences of unnecessary transfer and to develop interventions to reduce unnecessary strain on patients and their families, EMS, and hospital resources.

## METHODS

### Study Design and Data Source

We used a retrospective cohort study to analyze patient care and healthcare administrative data from a sample of pediatric patients who presented to the ED with asthma. The primary source of data for this study was the California Department of Health Care Access and Information (HCAI), from which we received a non-public version of the Emergency Department and Ambulatory Surgery (EDAS) Data and Patient Discharge Data (PDD) datasets. The HCAI compiles its data via mandatory standardized collection from all licensed hospitals throughout the state of California. The HCAI organizes data as unique encounters between a patient and healthcare facility, such that each record corresponds to one patient encounter at a given facility (eg, an interfacility transfer would generate two records for that patient). Visits to the ED that result in same-hospital admission are included in the PDD, whereas all other ED visits, including those that result in interfacility transfer, are included in the EDAS. Combining EDAS and PDD for a given year provides a full dataset of all unique, unduplicated ED visits in California within that year. The HCAI datasets are subject to standardized quality assurance procedures. More detailed information about HCAI can be found on its website.^15^

This study followed the Strengthening the Reporting of Observational Studies in Epidemiology (STROBE) reporting guideline for cross-sectional studies^16^ and was approved by the Mass General Brigham Human Research Committee and the California Committee for the Protection of Human Subjects.

### Study Population

We extracted all ED visits from the EDAS and PDD datasets from HCAI during 2016–2019. We included all patients aged 2–17 years to specifically study pediatric patients, and we excluded patients younger than two years of age because wheezing at this young age is more likely attributable to a transient condition such as bronchiolitis rather than a chronic one such as asthma.^17^^,^^18^ We included pediatric patients who presented to the ED and had a diagnosis of asthma (ie, International Classification of Diseases, Rev 10, codes J45, J98.01, and R06.2) for any documented discharge diagnosis. Finally, we included pediatric asthma ED visits that resulted in an interfacility transfer from the ED, regardless of the initial care setting at the receiving facility (eg, ED to ED, ED to inpatient floor, ED to intensive care unit, etc). In summary, the final study sample included patients aged 2–17 years who presented to an ED in California during 2016–2019, were diagnosed with asthma, and were transferred via EMS to another facility.

### Measures

The primary outcome measure used for this study was potentially unnecessary interfacility transfer. This measure was designed to capture patient transfers that—within the limitations of this data source—were not associated with clear retrospective indications that the patient received clinically necessary services that required transfer. We defined potentially unnecessary pediatric transfer based on recent literature (including all chief complaint categories),^9^ where the transfer did not result in a disposition of death or admission >24 hours, involve sedation or advanced imaging (defined as any imaging study apart from plain radiographs), or incur operating room or critical care charges. We added respiratory therapy as an additional marker of necessary transfer in the case of asthma, in addition to services captured under critical care such as positive-pressure ventilation. All remaining transfers were considered necessary.

More generally, we identified interfacility transfers by finding two encounters associated with a single unique patient identifier within one day of each other per encounter date, where the encounters occurred at two separate hospitals, and where disposition at the sending facility was designated as a transfer. Additional variables of interest included sociodemographic characteristics and details to describe patients’ medical history and healthcare utilization related to asthma. Demographic data included patient age, gender, race, ethnicity, primary language, residence urbanicity, and insurance status. We also included the number of ED visits and hospital admissions each patient had per year where asthma was listed as the discharge diagnosis. Finally, we calculated the number of complex chronic conditions from each patient’s past medical history per the Pediatric Complex Chronic Condition version 2 system (including technology dependence and organ transplantation).^19^^,^^20^

### Statistical Analysis

First, we used descriptive statistics to compare pediatric transfers for asthma that met vs did not meet criteria for potentially unnecessary transfer. Comparisons between groups were made using *t*-tests, Wilcoxon rank-sum tests, or chi-square tests as appropriate. We used generalized estimating equations (GEE) accounting for clustering by facility to calculate adjusted odds of unnecessary transfer, estimated with binominal distribution, logit link function, working independence correlation, and robust standard errors. Covariates included the patient-related factors described above that were included a priori based on prior literature and substantive reasoning. We performed all statistical analyses using Stata version 15.0 (StataCorp, College Station, TX).

## RESULTS

**Figure 1.**
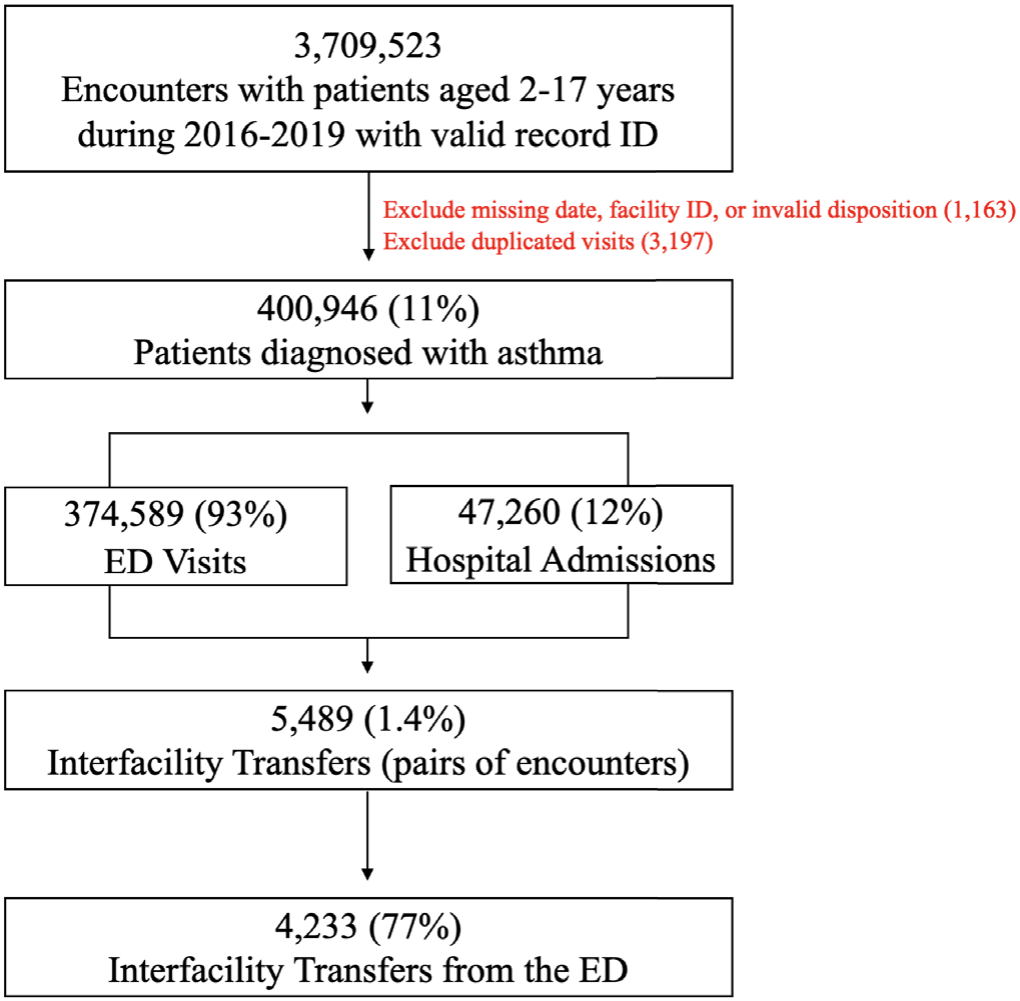
Inclusion and exclusion criteria used to develop the final study sample. Statistics regarding emergency department visits and hospital admissions do not represent patients being included or excluded; instead they provide context to aid in understanding the relative rate of patient transfers within the sample. *ED*, emergency department.

From an initial sample of 3,709,523 pediatric encounters, a total of 4,233 patients with asthma were transferred from an ED (each including a pair of two encounters, one at the sending facility and a second at the receiving facility; Figure 1). Among this sample, 461 (11%) met criteria as potentially unnecessary. The mean age of all pediatric patients with a transfer for asthma was 10.8 years (SD 4.8), 47% were female, and 41% were Hispanic (Table 1). Patients with a potentially unnecessary transfer were younger (9 vs 11 years, *P* < 0.001), more often male (65% vs 53%, *P* < 0.001), and more often had Hispanic ethnicity (51% vs 40%, *P* < 0.001). In terms of clinical characteristics, patients who met criteria for potentially unnecessary transfer less often had a complex chronic condition (8% vs 11%, *P* = 0.05) and over the prior year experienced fewer ED visits (1.6 vs 2.1, *P* < 0.001) and hospital admissions (0.6 vs 1.2, *P* < 0.001) for asthma.

**Table 1.** Descriptive characteristics and comparison of necessary and potentially unnecessary interfacility transfers.

Factor	Overall	Necessary	Unnecessary	*P*-value^*^
Total n	4,233	3,772 (89)	461 (11)	
Age in years				
Mean (SD)	10.8 (4.8)	11.0 (4.8)	9 (4.6)	<0.001
Age category, n (%)				<0.001
Child (2–5 years)	843 (20)	718 (19)	125 (27)	
School age (6–12 years)	1,451 (34)	1,249 (33)	202 (44)	
Teen (13–17 years)	1,939 (46)	1,805 (48)	134 (29)	
Gender, n (%)				<0.001
Female	1,952 (46)	1,789 (47)	163 (35)	
Male	2,281 (54)	1,983 (53)	298 (65)	
Race/ethnicity, n (%)				<0.001
Non-Hispanic White	1,041 (25)	961 (26)	80 (17)	
Non-Hispanic Black	918 (22)	835 (22)	83 (18)	
Hispanic	1,724 (41)	1,491 (40)	233 (51)	
Non-Hispanic other	518 (12)	455 (12)	63 (14)	
Missing	*32*	*30*	*NR*	
Primary language, n (%)				0.02
English	3,874 (92)	3,467 (92)	407 (88)	
Spanish	322 (8)	275 (7)	47 (10)	
Other or missing	37 (1)	30 (1)	NR	
ED visits without transfer for asthma per year				
Mean (SD)	2.1 (1.9)	2.1 (2.0)	1.6 (1.6)	<0.001
>2 ED visit per year, n (%)	1,068 (25)	983 (26)	85 (18)	<0.001
Admissions for asthma per year				
Mean (SD)	1.2 (0.8)	1.2 (0.8)	0.6 (0.7)	<0.001
>1 admission per year, n (%)	688 (16)	669 (18)	19 (4)	<0.001
Any complex chronic condition^†^, n (%)	463 (11)	425 (11)	38 (8)	0.050
Patient residence urbanicity, n (%)				0.15
Rural	83 (2)	78 (2)	NR	
Urban	4,131 (98)	3,679 (98)	452 (99)	
Missing	*19*	*15*	*NR*	
Insurance status, n (%)				0.85
Public	2,514 (59)	2,235 (59)	279 (61)	
Private	1,548 (37)	1,385 (37)	163 (35)	
Self-pay/other/missing	171 (4)	152 (4)	19 (4)	

Column percentages shown. Percentages may not sum to 100 due to rounding.

^*^ *T*-test or chi-square test as appropriate.

^†^ Complex chronic conditions defined using version 2 definition from Feudtner et al, 2014, (https://bmcpediatr.biomedcentral.com/articles/10.1186/1471-2431-14-199), adapted from Kurowski et al, 2014. (https://pubmed.ncbi.nlm.nih.gov/25039935/). Notably, the version 2 definition includes technology dependence and transplant but does not add these to the total. In this analysis, technology dependence and transplant are included in total complex chronic condition.

*ED*, emergency department; *NR*, not reported (due to data reporting restrictions).

Using the GEE model to account for clustering by hospital (Table 2), we found that younger age groups were associated with increased adjusted odds of potentially unnecessary transfer (ie, age 2–5 years, odds ratio [OR] 2.01, 95% confidence interval [CI] 1.39–2.91, compared to age 13–17 years). Male gender (OR 1.39, 95% CI 1.15–1.70) was also associated with increased odds of potentially unnecessary transfer. No associations were found with insurance status, residence urbanicity, or primary language. Patients of Hispanic ethnicity, compared to non-Hispanic White patients, had increased odds of potentially unnecessary transfer (OR 1.59, 95% CI 1.21–2.10). (Note that Hispanic ethnicity was used as the referent in Table 2 given that this was the largest racial/ethnic group in this sample.) Two or more hospital admissions for asthma per year was associated with decreased odds of potentially unnecessary transfer (OR 0.22, 95% CI 0.13–0.36), whereas no associations were found with ED visits for asthma or absence of any complex chronic conditions.

**Table 2.** Odds of unnecessary transfer using generalized estimating equations to account for clustering by hospital.

Factor	AOR	95% CI
Age category		
Child (2–5 years)	2.01	1.39, 2.91
School age (6–12 years)	2.03	1.50, 2.73
Teen (13–17 years)	1.00 (referent)	
Gender		
Female	1.00 (referent)	
Male	1.39	1.15, 1.70
Race/ethnicity		
Hispanic	1.00 (referent)	
Non-Hispanic Black	0.63	0.45, 0.86
Non-Hispanic White	0.63	0.48, 0.83
Non-Hispanic other	0.88	0.65, 1.20
Primary language		
English	1.00 (referent)	
Spanish	1.18	0.82, 1.70
Other or missing	1.69	0.74, 3.87
ED visits for asthma per year		
0–2	1.00 (referent)	
≥3	0.94	0.68, 1.28
Admissions for asthma per year		
0–1	1.00 (referent)	
≥2	0.22	0.13, 0.36
Complex chronic condition^*^		
None	1.00 (referent)	
≥1	0.79	0.55, 1.15
Patient residence urbanicity		
Rural	0.72	0.35, 1.58
Urban	1.00 (referent)	
Insurance status		
Public	0.98	0.75, 1.28
Private	1.00 (referent)	
Self-pay/other/missing	1.02	0.55, 1.89

^*^ Complex chronic conditions defined using version 2 definition. In this analysis, technology dependence and transplant are included in total complex chronic condition.

*AOR*, adjusted odds ratio; *CI*, confidence interval; *ED*, emergency department.

## DISCUSSION

Using a comprehensive, statewide dataset of ED visits and admissions, we found several patient characteristics associated with potentially unnecessary transfer of pediatric patients who present to the ED with asthma. These findings describe the patient-level characteristics associated with elevated (or reduced) odds of potentially unnecessary transfer, which can inform policy makers and ED administrators to consider subpopulations with elevated risk of unnecessary transfer when developing future studies and policies related to the transfer of pediatric patients with asthma. Potentially unnecessary transfers mark cases where patients do not show evidence of the benefits of transfer, such as a higher level of care or access to a specialist but do experience the risks and costs associated with transfer.

The rate of potential unnecessary transfer among this cohort was 11%, which is much lower than reported previously in studies of undifferentiated pediatric patients, including rates of one-in-two to nearly nine-tenths.^9^^,^^21^^,^^22^ However, in prior literature the diagnostic category of respiratory emergencies had the greatest number of transferred pediatric patients and was the only diagnostic category associated with decreased odds of direct discharge home from the ED, which may at least partially explain why we observed a lower rate of potentially unnecessary transfer.^21^ Perhaps respiratory emergencies are relatively less likely to be quickly discharged from the receiving facility compared to other diagnoses because they are more likely to involve an observation period (eg, continuous oxygen saturation monitoring, gradual reduction in frequency of respiratory treatments), or perhaps because emergency physicians have more comfort with decision-making for these transfers due to their more commonplace nature. Similarly, the increased prevalence of observation units may have contributed to this finding compared to earlier studies when this option was less available.

Demographic factors associated with increased odds of unnecessary transfers included younger age, male gender, and Hispanic ethnicity. Younger patients were associated with increased odds of potentially unnecessary transfer, which was consistent with prior reports that found particularly high risk among preschool-age patients.^9^^,^^22^ One possible explanation for this finding could simply be relatively inferior comfort among clinicians caring for younger patients. Increased odds of potentially unnecessary transfer among male patients was not expected a priori and was not found in the literature among undifferentiated pediatric patients, where female patients are more often found to be at higher risk when gender-based differences are found.^9^ Prior research has suggested that male pediatric patients tend to have greater prevalence and illness severity of asthma before puberty, in contrast to greater prevalence and severity among females after puberty, with some indication that sex hormones may play a role.^23^^,^^24^ Further research focused on the transfer of pediatric patients with asthma will be needed to determine whether gender-related differences are widespread, and if so, what may account for this disparity.

Hispanic ethnicity was also found to be associated with increased odds of potentially unnecessary transfer. One possible contributing factor to this finding might have been language barriers; however, even after controlling for primary language spoken, we found an independent association with Hispanic ethnicity. Hispanic ethnicity has previously been reported to be associated with increased odds of unnecessary transfer, but the reasons for this remain unclear.^9^^,^^25^ Given the known financial costs and medical risks that can be associated with interfacility transfer^7^^,^^8^ and prior findings that Hispanic patients incur greater costs associated with their asthma-related care in general,^26^ we encourage further work to evaluate this trend to gain a better understanding of how to minimize disparities in the burden of undue risk associated with potentially unnecessary interfacility transfer.

We found no association with patient residence urbanicity or with insurance status, in contrast to prior research that found increased risk of potentially unnecessary transfer among urban patients and those with public health insurance.^9^ In contrast to our findings, important prior work has highlighted that rural patients are more likely to experience potentially unnecessary transfer because their nearest hospitals tend to be less resourced, especially for pediatric care, compared to urban patients who are more likely to reside in geographic proximity to more resourced centers that are less likely to transfer pediatric patients.^8^ Characteristics specific to the state of California may account for these differences, although rates of public insurance and urban residence were quite similar in this cohort compared to prior work. Alternatively, our focus on asthma could be the explanatory factor, given that asthma is more common in urban settings (particularly in the presence of other factors that tend to affect such areas, such as environmental and housing-related insults) and is considered an ambulatory care-sensitive condition shown to be modulated by changes in insurance status among populations.^14^^,^^27^ Taken together, a solid, generalizable conclusion regarding the potential association between potentially unnecessary transfer and urbanicity or insurance status remains difficult to reach.

Finally, regarding clinical patient-level factors, we found that multiple hospital admissions for asthma per year were associated with decreased odds of potentially unnecessary transfer, whereas no association was found with ≥3 ED visits or the presence of a complex chronic condition. The finding that multiple hospital admissions for asthma was associated with decreased risk is not unexpected. The increased risk of mortality associated with recent hospitalization for asthma not only suggests greater likelihood of increased clinical severity among this cohort but is also a well documented and widely taught piece of evidence that directly factors into clinical decision-making, which makes it less likely for such patients to meet criteria for potentially unnecessary transfer.^28^^,^^29^ However, the same line of work that tends to indicate the association between hospitalizations and mortality often highlights similar associations with ED use, albeit an intuitively weaker association given that children who visit the ED and are admitted presumably have more severe asthma than those who are instead discharged.

Prior research has shown that complex chronic conditions tend to be associated with longer length of stay and greater resource utilization when transferred compared to not transferred for admission, suggesting that complex pediatric patients tend to be appropriately transferred. However, we did not find decreased risk among this cohort, perhaps due to over-triage in some cases where an asthma exacerbation was relatively mild, but transfer was nevertheless pursued out of concern for poor reserve and likely clinical deterioration or to provide a higher level of specialized care, perhaps in some cases with teams to whom the patient is known.

## LIMITATIONS

There are limitations associated with this study. First, the HCAI data includes a mix of administrative and clinical data sourced from patient health records. Thus, definitions of key variables, such as race/ethnicity and even including the diagnosis of asthma, are subject to information bias and misclassification. Most notably, the primary outcome measure uses a composite definition previously established in the literature but slightly adapted for the purposes of a study focused on asthma. This definition of potentially unnecessary transfer is a useful tool for retrospective research but is far from perfect and likely includes some degree of error associated with misjudgment in the absence of more detailed case-by-case information. Moreover, appropriateness of discharge within 24 hours was not assessed, such as thorough documentation of return visits. Similarly, defining the inclusion criteria for this study relied upon assumptions and trade-offs, such as including patients with any active diagnosis of asthma exacerbation, rather than primary diagnosis, to overcome the limitations of this dataset despite the risk of including patients with other primary diagnoses.

Second, the HCAI dataset is restricted to the state of California. California is a large, heavily populated, and diverse state, which makes findings from samples of its residents more nationally relevant than those from most states. However, the generalizability of these findings to other states and nationally is unclear. Third, the models used in this analysis focus on patient-level characteristics, therefore neglecting the many other factors that have been reported to play into the decision for interfacility transfer, including patient volumes and hospital capacities, hospital-related factors such as resources available, and community-related factors such as the availability of outpatient physicians. Fourth, we used a retrospective cohort design, which limits the interpretation of these findings.

Fifth, data was from 2016–2019, prior to the COVID-19 pandemic. The COVID-19 pandemic had widespread and complex effects on the US healthcare system, including the management of respiratory conditions and the reallocation of pediatric and critical care resources throughout systems; thus, use of data prior to the pandemic might more closely relate to current conditions as the US healthcare system continues to adapt and further normalize. Ongoing research into the effects of the COVID-19 pandemic on the interfacility transfer of pediatric patients with respiratory complaints will provide additional information to better evaluate this assessment.

## CONCLUSION

Using a statewide dataset of ED visits and admission, we found that younger, Hispanic, and male children who presented to the ED with asthma had higher odds of experiencing potentially unnecessary interfacility transfer. Patients with multiple hospital admissions for asthma within the prior year were found to have decreased odds of potentially unnecessary transfer. Important next steps in this line of investigation include studies targeted at discrepancies between these findings and prior research, investigation of the financial costs associated with unnecessary transfer of pediatric patients with asthma, and analysis of the healthcare systems-related factors associated with potentially unnecessary interfacility transfers. These insights can be considered by policy makers and ED administrators to identify subpopulations of patients that are more likely to be impacted by new interventions or to inform future studies concerned with disparities in delays in care or financial costs associated with unnecessary transfer. Findings from this study will need validation through a more rigorous prospective study to confirm the patient characteristics that might be associated with increased risk of possibly avoidable transfers and the potential consequences associated with them.
